# Does the “17-year gap” tell the right story about implementation science?

**DOI:** 10.3389/frhs.2025.1704368

**Published:** 2025-11-17

**Authors:** Denise Thomson, Gabrielle L. Zimmermann, Stephanie Montesanti

**Affiliations:** 1 Alberta SPOR SUPPORT Unit, University of Alberta, Edmonton, AB, Canada; 2 Department of Community Health Sciences, Cumming School of Medicine, University of Calgary, Calgary, AB, Canada; 3 School of Public Health, University of Alberta, Edmonton, AB, Canada

**Keywords:** implementation, implementation science, implementation practice, speed, evidence-based practice

## Introduction

The challenges of implementing and sustaining evidence-based organizational and system changes have been well documented. Over the past three decades, the field of implementation science (IS)—the scientific study of methods and strategies facilitating the uptake of evidence-based practice and research into regular use by practitioners and policymakers—has emerged to support this work (1). IS is now a rich field with specialized journals that publish increasingly sophisticated, multidisciplinary research (2). Scholars have developed theories, models, and frameworks (TMFs) based on insights from multiple disciplines to address the complex range of factors affecting the uptake of interventions. The value of these TMFs has been recognized in implementation planning related to pressing issues such as adapting to the health impacts of climate change, eradicating polio, and addressing global health inequity (3–5).

We—the three authors of this commentary—are researchers and practitioners of IS. In these roles, we have observed IS being applied across a wide range of public health and healthcare implementation processes in our home province of Alberta, Canada. We have noticed that funders, research teams, and health system teams often justify the importance of IS-based planning by stating that there is a “17-year gap” between the generation of research evidence and its application in practice and policy [see, e.g., Canadian Institutes of Health Research (6)]. This figure originates from an article by Balas and Boren published in the year 2000 (7). The concept of this gap is widely prevalent in the IS literature. While writing this commentary, we checked PubMed and found that the Balas and Boren article had been cited 2,237 times by that point (April 2025). We retrieved the 135 English-language articles published in 2024 and early 2025 that cited this paper and classified them according to how the article was cited. Over half (*n* = 77) of the author teams cited the article with specific reference to the 17-year gap, using language such as the lag time of adoption of new evidence-based treatments is “currently estimated to be approximately 17 years” (8), “It takes about 17 years for research evidence to get to clinical practice” (9) or, “Successful translation from scientific discoveries to implementation in clinical practice and public health takes on average 17 years” (10). A further 28 author teams cited the paper in support of the premise that implementation takes an excessively long time, without explicitly citing the 17-year figure. The often-unstated assumptions behind quoting this article are that, first, 17 years is too long a time frame for realizing the benefits of research, and, second, “putting evidence into practice” is a straightforward concept that does not require more nuanced conceptualization.

In this commentary, we argue that incorporating references to the 17-year gap in articles and presentations, rather than illuminating the need for IS, actually obscures the present state of the field and the challenges of cocreating and sustaining change in complex systems (11).

## Arguments

### Argument 1: the citation does not support the 17-year figure

The Balas and Boren article cited in support of the 17-year gap actually presents a much more nuanced picture of the time it takes to move research into practice—and of measuring what moving research into practice actually means. The authors first present the findings of several studies of the time taken to move evidence into practice; the average time was 17 years, but the figures in the included studies varied by clinical specialty and by the definition of what “moving evidence into practice” actually means. The second half of the paper presents the authors’ own analysis of the time required across nine clinical disciplines to achieve a 50% rate of clinical use of findings from a landmark study. They found an average annual increase of 3.2% across nine clinical areas, which gave an average of 15.6 years from publication of the landmark study to a 50% utilization rate. This was broken down into 6.3 years for evidence to reach reviews, papers, and textbooks and 9.3 years to implement the findings into practice. In short, what this paper supports is not the blanket statement “we know it takes 17 years,” but, in reality, a much more nuanced, context-sensitive picture in which the time required to move evidence into practice varies according to clinical specialty, implementation fidelity, local context, and the chosen benchmark.

### Argument 2: 25-year-old evidence is not relevant in today's world

British novelist L. P. Hartley famously wrote, “The past is a foreign country; they do things differently there” (12). Implementation scientists think long and hard about the importance of context. The context of 2025, in terms of factors influencing diffusion and implementation, is vastly different from that of 2000. When Balas and Boren's article was published, the first iPhone had yet to come on the market, Netflix still made its money snail-mailing DVDs to customers, and social media as we currently know it, with its power to spread messages far and wide, did not exist—Facebook was launched in 2004 and Twitter in 2006. Jonathan Lomas’ landmark editorial on what was then called “knowledge transfer” had only just been published (13), the concept of health-related knowledge brokerage was in its infancy, and the publication dates of important implementation science frameworks such as the Consolidated Framework for Implementation Research and the Exploration, Preparation, Implementation, and Sustainment framework were years in the future (2009 and 2011, respectively). In 2025, implementers have dozens of IS TMFs to choose from (14). Furthermore, we are benefiting from the rise of numerous implementation support structures and specialists. Two Canadian examples, both supported by funding from the Canadian Institutes of Health Research, are the Health System Impact Program, which provides embedded research opportunities for PhD students, postdoctoral fellows, and early career researchers, and the provincial and territorial SPOR SUPPORT Units, which provide local decision-makers and health system staff with support for learning health systems, such as improved data access and implementation science expertise. Many countries are supporting initiatives such as partnerships between academic and healthcare organizations, embedded researcher positions, and intermediary organizations and programs, all of which create preconditions for accelerating implementation.

### Argument 3: we have not established the optimal pace of implementation

If 17 years is too long to implement change, what is the right time frame? Somewhat surprisingly, until recently, there has been little research on the optimal pace of implementation and the factors that affect its speed, including changing political environments, organizational readiness, and the capacity of systems (15). Intervention characteristics and implementation context matter. Speedy implementation is more likely for low-risk interventions supported by strong evidence and/or in contexts with high readiness and where important relational work has been done (15). Key components such as building trust and credibility between partners, attending to health equity, and identifying local champions—work that helps sustain interventions and programs in practice—all take time (16). In addition, “strategic delay” of implementation may sometimes allow space for clarification and necessary adaptations that will improve quality and sustainment in the long run (17). Moreover, faster may not always be better; while the COVID-19 pandemic saw accelerated production and uptake of new evidence, it also highlighted the dangers of implementing unproven cures, such as ivermectin, without a mature evidence base. The field of IS will benefit from more work on the challenges and uncertainties associated with accelerating implementation efforts, with attention paid to the fact that implementation and sustainment are often slower in disadvantaged, security-challenged, or mistrustful communities or in settings with under-resourced health systems (15, 18).

### Argument 4: we have a much more interesting story to tell now

In a resource-constrained world with pressing social ills, every failed implementation is a missed opportunity to benefit people and communities. Implementation science grew out of the recognition that successful implementation and sustainment are hard work. There is no straight line between designing a policy or program and seeing it taken up by the people, organizations, and systems that will make the necessary changes happen. As Rapport et al. note, “what appears to be a linear process is contested, challenging, tortuous, and political—governed more by the laws of complexity and chaos than those inherent in straight-line, formulaic models” (19). With this understanding, justifying our work with statements about 17 years to “move evidence into practice” masks the complexity of the research–practice ecosystem.

## Discussion

We have presented our case for why researchers and practitioners of IS may wish to reconsider citing the 17-year gap as a justification for the field. The article cited in support of this time frame is now 25 years old and reflects a world that no longer exists. Supporting the adoption of evidence-based interventions is not a linear process—the factors that accelerate or inhibit implementation of any given initiative are influenced by a wide range of contextual factors within complex research–practice ecosystems. IS theories, models, and frameworks largely originated in affluent Western settings. In recent years, however, innovative scholarship has pushed the field to be more reflective of global health. To cite just two of many possible examples, Harding and Oetzel have developed the He Pikinga Waiora implementation framework to support the analysis of implementation effectiveness in Indigenous communities in Aotearoa New Zealand (20), and Means et al. have suggested a modification of Consolidated Framework for Implementation Research (CFIR)—adding the domain of characteristics of systems—to better reflect the decentralized nature of health systems in many low- and middle-income countries (LMICs) (21). Given this evolution, citing the supposed 17-year delay between the generation and the application of evidence, based on work conducted many years ago, no longer reflects the complexity, nuance, or evolution of our field.

We stated earlier that authors cite the 17-year figure as a pithy and readily understood justification for IS—a statement easily incorporated into articles, reports, and presentations. Readers of this article, convinced (we hope) by our argument, may justifiably wonder if we have an alternative to propose. We suggest an approach that underscores the potential of IS to improve people's lives in all parts of the globe, quoting the United Nations’ Universal Declaration of Human Rights, Article 27, that, “Everyone has the right … to share in scientific advancement and its benefits” (22). We read this as not only a call for equitable access to the benefits of science but also a call for a more equitable recognition of the voices and knowledge that constitute science around the world. In other words, the ultimate aim of implementation is not speed alone but ensuring that scientific knowledge is mobilized to advance health and wellbeing for all.

## Concluding remarks

To ensure that all people derive benefits from research, a careful design of implementation plans that include strategies and mechanisms robust enough to support the promise of data-informed learning health systems is required (23). If, after 25 years, the 17-year figure is still routinely cited, then we must ask ourselves whether implementation science is truly bridging the evidence–practice gap or simply reinscribing it. We call on scholars, practitioners, and educators to reconsider the 17-year trope and instead illuminate the real and diverse challenges of implementation—challenges that require thoughtful, context-sensitive, and time-responsive approaches—drawing on current scholarship reflecting global perspectives.
